# Problematic usage of the internet is associated with orthorexia nervosa tendency in adults

**DOI:** 10.1017/S1092852925100771

**Published:** 2025-12-12

**Authors:** Sophie Boutouis, Jon E. Grant

**Affiliations:** Psychiatry and Behavioral Neuroscience, The University of Chicago Medicine, USA

**Keywords:** Internet addiction, internet use, orthorexia, orthorexia nervosa, eating disorder

## Abstract

**Background:**

The link between problematic usage of the internet (PUI) and eating disorder symptoms is well-established. However, less is known about this association in the context of orthorexia nervosa (ON), an excessive preoccupation with healthy eating. This study aimed to investigate the relationship between PUI, including various problematic online behaviors, and ON tendency in a sample of US adults.

**Methods:**

Three hundred adults completed an online survey via prolific (mean age = 37.40, 54.6% female). The survey included demographic questions, the ORTO-R to measure ON symptoms, and the Internet Severity and Activities Addiction Questionnaire (ISAAQ-10) to assess PUI and the extent of engagement in several online activities, such as gaming, shopping, social networking, cyberchondria, pornography, and cyberbullying.

**Results:**

Women had higher ORTO-R scores than men (*p* < .05). ISAAQ-10 scores predicted ORTO-R scores in a regression model (*β* = .375, *p* < .001) even after controlling for age, gender, and an eating disorder diagnosis. ON risk was associated with a high engagement in cyberchondria, researching healthy food choices on the internet, cyberbullying perpetration, and online shopping.

**Conclusions:**

Results indicate that PUI is associated with ON symptoms even after considering other predictors. Maladaptive use of nutrition and medical-related resources may play a significant role in this association. However, it remains unclear whether exposure to this content increases ON risk or if people with ON symptoms seek this information online. The directionality of this relationship is an important area for future research.

## Introduction

Orthorexia nervosa (ON) is a proposed eating disorder characterized by an intense preoccupation with healthy eating and a fixation on food quality and purity.^1^ Individuals with ON follow rigid dietary rules, often eliminating entire food groups deemed unhealthy from their diets and spending an excessive amount of time planning, obtaining, and preparing their food.^2^ This level of restriction results in marked distress and psychosocial or medical impairments that disrupt overall functioning.^2^ For instance, individuals with ON may experience significant anxiety in social situations that involve food and thus opt to avoid these events altogether.^3^ They may also distance themselves from family and friends who do not adhere to their self-imposed dietary rules, leading to further isolation.^4^ ^,^^5^ While ON is gaining recognition in the literature and media, it has yet to be established as a disorder in the Diagnostic and Statistical Manual of Mental Disorders (DSM) or the International Classification of Diseases (ICD).

The occurrence of ON varies across populations: in the general population, the rate of ON may range from 1 to 57.6%,^6^ ^,^^7^ but in high-risk groups, such as athletes or health professionals, estimates range from 35 to 57.8%.^8^ However, it must be noted that some of these studies measured ON symptoms with the ORTO-15, an instrument that has been increasingly criticized for its poor reliability and validity.^9^ ^,^^10^ This measure fails to distinguish between pathological and non-pathological healthful eating^1^ but continues to be used in ON research, resulting in questionable results. This issue, coupled with the overall lack of consensus on diagnostic criteria for ON, makes it difficult to determine the prevalence of this complex behavior.

One aspect that researchers *can* agree on regarding ON is that the internet may play a significant role in its development and/or maintenance.^11^ Problematic usage of the internet (PUI)—also known as internet addiction—is an umbrella term referring to excessive engagement in online activities that results in significant psychosocial and occupational impairments.^12^ PUI encompasses a variety of behaviors, including gaming, shopping, gambling, social networking, engaging in cyberchondria (the repeated seeking of medical information to alleviate anxiety or distress) or pornography, streaming media, and cyberbullying.^13^ Internet addiction has been linked to obsessive-compulsive^14^ and eating disorder symptoms,^15^ ^,^^16^ which have significant theoretical overlap with ON.^17^ As such, PUI may also be associated with an ON risk.

Several studies have connected a high level of involvement with nutrition and fitness accounts on visual-based social media platforms (particularly Instagram) with ON symptoms.^18^ ^–^^20^ An analysis of “healthy” recipes posted on Instagram found that most of them were unbalanced and did not meet nutritional guidelines.^21^ The link between social media use and ON may be mediated by the drive for thinness and muscularity,^18^ attitudes toward eating,^22^ and obsessive-compulsive symptoms.^23^ Additionally, social media addiction has been positively correlated with ON symptoms.^20^ ^,^^24^

There is a growing concern, however, about how other forms of PUI (outside of social media use) may affect the development and maintenance of eating disorders. Indeed, previous research has connected compulsive shopping,^25^ ^,^^26^ excessive pornography use,^27^ ^,^^28^ and cyberbullying perpetration^29^ with eating disorder symptoms. In the context of ON, 2 studies have examined its relationship with cyberchondria. Atsizata and Cangöl Sögüt^30^ found that among nurses, cyberchondria increased their risk of ON. However, in a sample of pregnant women, cyberchondria and ON levels were unrelated.^31^ This relationship in a community sample is unclear.

Additionally, no studies have examined the associations between “global” internet use problems or other online activities and ON. These gaps in the literature are important to fill because 96% of US adults use the internet,^32^ and various facets of internet usage overlap. For instance, a recent review supported the interaction of several digital hazards, including cyberbullying, exposure to pro-eating disorder content on social media, and online gaming, with preexisting risk factors (eg, thin-ideal internalization) to predict eating disorder symptoms and clinical manifestations of eating disorders.^33^ In the case of ON, someone may excessively seek nutritional information on social media and engage with fitness accounts that post oversexualized content while leaving harmful comments or bullying other users. These aspects of PUI may have distinctive influences on orthorexic symptoms. As such, the current study aimed to determine the association between PUI (including gaming, shopping, social networking, cyberchondria, pornography, and cyberbullying) and ON tendency in a large, non-clinical sample of US adults. We also investigated demographic correlates of ON to add to the growing ON literature. Based on existing research, we hypothesized that PUI would predict ON risk, and a high engagement in social networking (particularly health-related content) and cyberchondria would be more strongly associated with ON tendency than other facets of PUI.

## Methods

### Participants

A total of 300 adults were recruited to complete an online survey about online behaviors and mental health via Prolific. Subjects had to be 18 years of age or older, currently residing in the United States, able to understand English, and capable of providing informed consent to participate. Participants were excluded if they were unable to understand or undertake study procedures.

### Procedures

Subjects completed an online survey via REDCap. They were first required to review the Institutional Review Board (IRB)-approved informed consent, in which they could accept or deny participation in the survey. The survey asserted that all responses would be kept confidential and that no personally identifying information would be collected. Participants were compensated $12 for their participation. Data was collected on 01/09/2024.

### Measures

The online survey collected demographic information (age, gender, race, etc.) and mental health history. Subjects were asked if they had ever been diagnosed with an eating disorder (including anorexia nervosa, bulimia nervosa, or binge eating disorder) or obsessive-compulsive disorder, as ON has several similarities to these conditions.^17^ Data on active substance and psychotropic medication use were also collected, with current nicotine or alcohol use considered as any use within the past 30 days.

To assess ON symptoms, all participants completed the ORTO-R. The ORTO-R is a revised version of the ORTO-15 that consists of 6 questions (selected from the original scale) that best reflect thoughts and behaviors associated with ON.^34^ The new version of the scale was created based on a reassessment of the original data used to develop the ORTO-15, and it appears to be a more reliable tool^34^ that has been validated in several studies of diverse populations.^34^ ^–^^37^ The 6 items assess the core features of ON, including an excessive preoccupation with healthy eating, a significant effort to eat healthily, food-related emotional distress, and impairments in social functioning.^34^ The ORTO-R measures ON tendency in a dimensional manner, eliminating a categorical diagnosis. Subjects were asked to respond to each question on a scale of 1 = Never to 5 = Always (eg, Are your rigid and restrictive dietary choices conditioned by your worry about your health status?). Higher scores indicate more severe ON symptoms. The ORTO-R showed good reliability in the present study (*α* = 0.809).

Participants also completed the Internet Severity and Activities Addiction Questionnaire (ISAAQ-10), which consists of 2 components that evaluate the severity of internet use and engagement in various online activities, respectively. In the first component, subjects were asked to respond to each question on a scale of 0 = Not at all to 5 = All the time (eg, How often do you find yourself losing track of time while engaging in an internet-related activity?). Higher scores indicate greater problematic internet use, with Ioannidis et al.^38^ proposing a cutoff score of 30 out of 50 as indicating “high PUI.” The ISAAQ-10 demonstrated excellent reliability in the present study (*α* = 0.903). The second component (part B) prompted participants to indicate how often they engaged in certain online activities during the last 6 mo. These activities included browsing or surfing the web, online gaming with other players, online shopping, social networking, researching medical information (cyberchondria), engaging in cybersex or viewing pornography, streaming music or videos on a platform, and cyberbullying others. The same scale (0 = Not at all, 1 = Rarely, 2 = Occasionally, 3 = Frequently, 4 = Very often, 5 = All the time) was used. Histograms revealed that subjects’ responses to most of the questions in part B clustered around the middle values of the scale. Based on the normal curves, participants were divided into 2 groups (those who chose values greater than or less than the means) to ensure that adequate sample sizes were used in the analyses.

To explore potential expressions of ON-like behaviors on the internet, subjects were asked the following yes or no questions:

1. Do you often search for information about healthy foods (eg, research nutritional information of the food you eat, look for healthy recipes) on the internet?
2. Do you often eat while engaging in an internet-related behavior?

### Statistical analysis

The data were analyzed using IBM SPSS Version 29.0. Between-group differences in continuous variables were tested using independent samples *t*-tests, analyses of variance (ANOVAs), or analyses of covariance (ANCOVAs) while controlling for covariates. Effect sizes were calculated in the form of Cohen’s *d* for *t* tests (0.2 = small, 0.5 = medium, 0.8 = large) and eta squared and partial eta squared for ANOVAs and ANCOVAs, respectively (0.01 = small, 0.06 = medium, 0.14 = large). Pearson correlation analyses were performed to assess the association between continuous variables. The robustness of significant correlations was probed by a multiple linear regression. A two-sided *p*-value of <0.05 was considered statistically significant in all analyses.

### Ethics

The Institutional Review Board of the University of Chicago approved the study and the consent statement. The authors assert that all procedures contributing to this work comply with the ethical standards of the relevant national and institutional committees on human experimentation and with the Helsinki Declaration of 1975, as revised in 2008.

## Results

A total of 300 adults participated in the study. Ten subjects did not complete the ORTO-R and were thus excluded from the analyses. As such, the final sample consisted of 290 adults (*M_age_* = 37.40, *SD* = 12.61), including 155 women, 120 men, 9 transgender or non-binary individuals, and 6 people who did not report their gender.

Descriptive statistics of the sample are presented in Table 1. Most participants identified as white or Caucasian (69.2%), had a college degree (47.0%), were single (36.6%) or married or engaged (36.9%), identified as heterosexual (78.5%), and did not report active nicotine (82.0%) or psychotropic medication (76.0%) use. Respondents reported an average of 1.22 (*SD* = 1.49) diagnosed psychiatric conditions. The mean score on the ISAAQ-10 was 18.48 (*SD* = 9.29), and 12.8% of the sample met criteria for PUI.

**Table 1.** Descriptive Statistics for a Sample of 290 Adults

Variable name (total *n*)		*M (SD) or %*
Age (*n* = 287)		37.40 (12.61)
Gender (*n* = 284)	Male	42.3
	Female	54.6
	Other	3.2
Race (*n* = 286)	White or Caucasian	69.2
	Black or African American	14.0
	Hispanic or Latino	5.2
	Asian or Pacific Islander	3.5
	Other	8.0
Education (*n* = 287)	High school or less	13.2
	Some college	26.5
	College	47.0
	Graduate school	13.2
Relationship status (*n* = 287)	Single	36.6
	Dating	19.9
	Married or engaged	36.9
	Separated or widowed	6.6
Sexual orientation (*n* = 288)	Heterosexual	78.5
	Gay or lesbian	3.5
	Bisexual	12.5
	Other	5.6
Current nicotine use (*n* = 289)	No	82.0
	Yes	18.0
Current alcohol use (*n* = 287)	No	27.2
	Yes	72.8
Taking a psychotropic medication (*n* = 288)	No	76.0
	Yes	24.0
Number of diagnosed psychiatric disorders (*n* = 290)		1.22 (1.49)
Diagnosed with an eating disorder (*n =* 290)		2.1
Diagnosed with obsessive-compulsive disorder (*n* = 290)		5.2

Subjects scored an average of 14.52 (*SD* = 4.95) on the ORTO-R, with women scoring higher than men (*M* = 15.10, *SD* = 4.93 versus *M* = 13.71, *SD* = 4.90, respectively; *t*(273) = −2.324, *p* = .021). Those who reported taking a psychotropic medication had higher ORTO-R scores than those in the comparison group (*M* = 15.86, *SD* = 5.47 versus *M* = 14.06, *SD* = 4.69, respectively; *t*(286) = −2.664, *p* = .008). Additionally, participants who had been diagnosed with an eating disorder (*M* = 20.83, *SD* = 3.97), scored higher on the ORTO-R than those who did not report such a diagnosis (*M* = 14.38, *SD* = 4.89; *t*(288) = −3.209, *p* = .001). ORTO-R scores did not differ between subjects based on all other demographic characteristics, including race, education, relationship status, sexual orientation, nicotine use, alcohol use, or a history of obsessive-compulsive disorder (see Table 2).

**Table 2.** Differences in Adults’ Orthorexia Nervosa Tendency According to Their Descriptive Statistics

Variable name		ORTO-R score, *M (SD)*	*t* or *F*	*p*	Cohen’s *d* or eta squared
Gender	Male	13.71 (4.90)	−2.324	.021	−.283
	Female	15.10 (4.93)			
Race	White or Caucasian	14.39 (5.05)	1.004	.406	.014
	Black or African American	13.85 (5.08)			
	Hispanic or Latino	16.67 (3.11)			
	Asian or Pacific Islander	15.40 (5.64)			
	Other	14.35 (4.37)			
Education	High school or less	13.95 (5.09)	.407	.748	.004
	Some college	14.30 (4.99)			
	College	14.59 (4.61)			
	Graduate school	15.11 (5.68)			
Relationship status	Single	14.04 (5.10)	.903	.440	.009
	Dating	14.81 (4.98)			
	Married or engaged	14.87 (4.82)			
	Separated or widowed	13.37 (4.45)			
Sexual orientation	Heterosexual	14.23 (4.85)	.874	.455	.009
	Gay or lesbian	15.20 (3.85)			
	Bisexual	15.56 (5.45)			
	Other	14.94 (5.41)			
Current nicotine use	No	14.53 (4.90)	.347	.729	.053
	Yes	14.27 (5.10)			
Current alcohol use	No	14.41 (5.47)	−.270	.787	−.036
	Yes	14.59 (4.77)			
Taking a psychotropic medication	No	14.06 (4.69)	−2.664	.008	−.368
	Yes	15.86 (5.47)			
Diagnosed with an eating disorder	No	14.38 (4.89)	−3.209	.001	−1.324
	Yes	20.83 (3.97)			
Diagnosed with obsessive-compulsive disorder	No	14.42 (4.87)	−1.462	.145	−.388
	Yes	16.33 (6.10)			

Bolded characteristics indicate a statistically significant difference in variable distribution between groups (*p* < .05).

ORTO-R scores were higher in participants who did (*M* = 17.11, *SD* = 4.77) versus those who did not meet criteria for PUI (*M* = 14.16, *SD* = 4.91; *F*(1, 270) = 7.819, *p* = .006). Moreover, there were several significant findings in relation to online activities. First, subjects who shopped online “frequently to all the time” (*M* = 15.44, *SD* = 4.94) had higher ORTO-R scores than those who shopped online “occasionally to not at all” (*M* = 14.10, *SD* = 4.91; *F*(1, 278) = 5.210, *p* = .023). The same results were found regarding cyberchondria (*M* = 15.48, *SD* = 14.35 versus *M* = 14.35, *SD* = 5.06, respectively; *F*(1, 278) = 4.181, *p* = .042). Participants who often researched healthy food choices on the internet (*M* = 17.61, *SD* = 4.50) had higher ORTO-R scores than those in the comparison group (*M* = 13.04, *SD* = 4.47; *F*(1, 276) = 55.988, *p* < .001). This variable had the largest effect size (ηp^2^ = .169) on the dependent variable. Lastly, subjects who cyberbullied others to any extent (*M* = 17.05, *SD* = 4.31) scored higher on the ORTO-R than those who reported no cyberbullying (*M* = 14.37, *SD* = 4.95; *F*(1, 278) = 8.724, *p* = .003). There was no difference in ON tendency among participants with different rates of other online behaviors, including social networking (see Table 3).

**Table 3.** Differences in Adults’ Orthorexia Nervosa Tendency According to Online Behaviors While Controlling for Age and Gender

Variable name		ORTO-R score*M (SD)*	*F*	*p*	Partial eta squared
High PUI	No (*n* = 238)	14.16 (4.91)	7.819	.006	.028
	Yes (*n* = 36)	17.11 (4.77)			
Browsing or surfing the web	Frequently or less (*n* = 163)	14.39 (4.76)	.546	.460	.002
	Very often or more (*n* = 114)	14.85 (5.26)			
Online gaming	Rarely or less (*n* = 146)	14.42 (4.94)	.072	.789	.000
	Occasionally or more (*n* = 135)	14.68 (4.99)			
Online shopping	Occasionally or less (*n* = 187)	14.10 (4.91)	5.21	.023	.018
	Frequently or more (*n* = 95)	15.44 (4.94)			
Social networking	Occasionally or less (*n* = 119)	14.34 (4.82)	.054	.816	.000
	Frequently or more (*n* = 163)	14.70 (5.06)			
Cyberchondria	Occasionally or less (*n* = 232)	14.35 (5.06)	4.18	.042	.015
	Frequently or more (*n* = 50)	15.48 (4.36)			
Pornography	Rarely or less (*n* = 197)	14.38 (4.91)	2.44	.120	.009
	Occasionally or more (*n* = 84)	14.99 (5.08)			
Streaming online media	Frequently or less (*n* = 162)	14.72 (4.89)	1.05	.307	.004
	Very often or more (*n* = 119)	14.31 (5.07)			
Cyberbullying others	Not at all (*n* = 263)	14.37 (4.95)	8.72	.003	.030
	Rarely or more (*n* = 19)	17.05 (4.31)			
Researching healthy food choices online	No (*n* = 187)	13.04 (4.47)	55.988	<.001	.169
	Yes (*n* = 93)	17.61 (4.50)			
Eating while engaging in an online behavior	No (*n* = 143)	13.92 (4.94)	2.856	.092	.010
	Yes (*n* = 139)	15.20 (4.89)			

Bolded characteristics indicate a statistically significant difference in variable distribution between groups (*p* < .05).

Abbreviation: PUI, problematic usage of the internet

Age was negatively correlated with ISAAQ-10 scores (*r* = −.352, *p* < .001) and ORTO-R scores (*r* = −.155, *p* = .009), and ISAAQ-10 scores were positively correlated with ORTO-R scores (*r* = .411, *p* < .001). After controlling for age, gender (0 = male, 1 = female), and an eating disorder diagnosis (0 = no, 1 = yes) in a multiple linear regression, ISAAQ-10 scores were still associated with ORTO-R scores (*t* = 6.336, *p* < .001). While the overall utility of the model was significant (*F*(4, 260) = 16.505, *p* < .001, *R^2^* = .203), age and gender did not significantly contribute to the model (see Table 4).

**Table 4.** Multiple Linear Regression Evaluating Predictors of Orthorexia Nervosa Tendency

Predictors				
	Standardized coefficients *β*	*t*	95% CI	*p*
Age	−.031	−.518	−.058–.034	.605
Gender	.109	1.966	−.002–2.195	.050
ISAAQ–10 total score	.375	6.336	.140–.266	<.001
Eating disorder	.192	3.456	2.752–10.042	<.001

Bolded characteristics are statistically significant predictors of the outcome variable (*p* < .05).

Abbreviation: ISAAQ-10, Internet Severity and Activities Addiction Questionnaire.

## Discussion

This is the first study to investigate the relationship between PUI and various online activities and ON tendency. In our sample, the average ORTO-R score was 14.52. This is consistent with similar studies: Dell’Osso et al.^39^ reported a mean ORTO-R score of 12.726 in a sample of college students, and in a combined clinical and non-clinical sample, Vaccari et al.^37^ found an average ORTO-R score of 16.6. Additionally, we found that women had a higher ON tendency than men. While some studies did not detect gender differences in orthorexic tendencies,^40^ ^,^^41^ our results are corroborated by other studies.^30^ ^,^^39^ ^,^^42^ The increased risk of ON among women may be explained by the similarities between anorexia and orthorexia nervosa,^43^ as women are more likely to be diagnosed with anorexia nervosa than men.^44^ Indeed, we found that subjects with an eating disorder and those on psychotropic medications had higher ORTO-R scores than those in the comparison groups. These findings support the growing literature that ON may contain more features of an eating disorder than obsessive-compulsive disorder^45^ ^,^^46^ and may be comorbid with conditions that warrant medication usage.

While the mean ISAAQ-10 score in our sample (18.48) is slightly lower than what has been reported in the literature,^38^ the rate of PUI among our participants (12.8%) is the same as that in the global population.^47^ Additionally, we found that as subjects’ ages increased, their risk of internet addiction and ON decreased. This is consistent with previous research.^48^ ^,^^49^ ON tendency (measured via ORTO-R scores) was significantly higher in those with than those without PUI, even after controlling for age and gender. Moreover, while considering age, gender, and an eating disorder diagnosis as covariates, a multiple linear regression revealed that ISAAQ-10 scores were significant positive predictors of ORTO-R scores. The mechanisms of this association are unclear, but perhaps anxiety, compulsivity, or a need for control play a significant role, as one may control their diet to cope with other areas of their life that feel uncontrollable (eg, difficulties with regulating internet use). Nevertheless, our finding demonstrates that “global” internet use problems are associated with ON tendency, even after adjusting for potential confounders. This is a valuable contribution to the literature, which has predominantly focused on social media usage in relation to ON.

Interestingly, we did not detect a difference in ON symptoms between subjects who used social media at different frequencies. This supports the idea that ON risk may be more impacted by the type of content viewed on social media than the duration of use.^50^ Indeed, we found that among all the online activities examined in our study, those who often used the internet to research the nutritional information of their food or look for healthy recipes had the highest ORTO-R scores. The effect size of performing this activity often (*ηp* ^2^ = .169) on ORTO-R scores was large, suggesting a strong association. However, the directionality of this relationship is unclear. Does exposure to clean eating-related content increase one’s risk of ON, or do people with ON symptoms seek out this information online? Future research adopting a longitudinal design is needed to get at this question.

In a similar vein, participants who engaged in high levels of cyberchondria had more ON symptoms than those in the comparison group. This result replicates that of Atsizata and Cangöl Sögüt,^30^ who found that cyberchondria increased nurses’ risk of ON, in a large community sample of adults. This is concerning given that around 60% of U.S. adults use the internet to search for medical information,^51^ but most of it (including nutritional information) is incorrect and of poor quality.^52^ ^,^^53^ As more and more people turn to the internet, which has a considerable amount of misinformation, for guidance about their health, the risk of ON may increase. While again, the directionality of this association is uncertain, it underscores a need for more research on cyberchondria and ON in this digital age.

Apart from excessive engagement with health-related content, our study found that subjects who shopped online “frequently or more” and those who cyberbullied others had higher ORTO-R scores than those in the comparison groups. This is in line with previous research that has suggested overlap between compulsive shopping^25^ ^,^^26^ and cyberbullying perpetration^29^ with eating disorders. Indeed, one study found that low self-esteem, which predicts greater cyberbullying,^54^ also plays a role in the relationship between compulsive shopping and disordered eating.^55^ While low self-esteem is a theorized risk factor of ON,^56^ future research should investigate the mechanisms of the association between various facets of PUI and ON.

This study has some limitations. First, the ORTO-R measures ON symptom severity in a dimensional manner, so there is no cutoff score available to indicate ON. Thus, most of our analyses were limited to correlations, and the clinical significance of our results is unclear. Yet, since there are still no fixed diagnostic criteria for ON, our findings signify associations between internet addiction and ON within the current scope of research. Next, the cross-sectional design of this study limited our ability to make causal connections. In a previous longitudinal study, PUI was cross-sectionally correlated with but not longitudinally predictive of eating disorder symptoms when considering socio-contextual variables,^57^ so we emphasize that our results do not suggest that PUI may cause ON. Additionally, all data were self-reported, which may be inaccurate due to social desirability and recall biases. Data regarding psychiatric diagnoses and PUI, in particular, would have been best verified by a clinician-administered diagnostic interview. Lastly, while Prolific has been shown to yield high-quality data,^58^ ^,^^59^ participants on Prolific may be unrepresentative of the population. In our sample, we had an approximately equal division of genders, but the majority of our participants were Caucasian.

## Conclusion

Our results indicate a higher prevalence of ON symptoms among women, individuals who have been diagnosed with an eating disorder, and those who take at least one psychotropic medication. Additionally, we reveal an association between broad internet use problems (including but not limited to social media use) and ON tendency. PUI was still a significant predictor of ON tendency after controlling for age, gender, and a history of an eating disorder. PUI in the form of excessive engagement with health-related content may drive this association; however, other online activities, including cyberbullying and compulsive shopping, may also be involved and warrant more attention in the literature. Further research is needed to establish the directionality and mechanisms of these associations.
